# Recent Progress in Molecular Oxygen Activation by Iron-Based Materials: Prospects for Nano-Enabled In Situ Remediation of Organic-Contaminated Sites

**DOI:** 10.3390/toxics12110773

**Published:** 2024-10-24

**Authors:** Fangru He, Lianrui Xu, Hongyang Wang, Chuanjia Jiang

**Affiliations:** 1 College of Environmental Science and Engineering, Ministry of Education Key Laboratory of Pollution Processes and Environmental Criteria, Tianjin Key Laboratory of Environmental Remediation and Pollution Control, Nankai University, Tianjin 300350, China; 2 State Key Laboratory of Environmental Criteria and Risk Assessment, Chinese Research Academy of Environmental Sciences, Beijing 100012, China

**Keywords:** oxygen activation, Fe-based materials, reactive oxygen species, organic pollutants, groundwater contamination

## Abstract

In situ chemical oxidation (ISCO) is commonly used for the remediation of contaminated sites, and molecular oxygen (O_2_) after activation by aquifer constituents and artificial remediation agents has displayed potential for efficient and selective removal of soil and groundwater contaminants via ISCO. In particular, Fe-based materials are actively investigated for O_2_ activation due to their prominent catalytic performance, wide availability, and environmental compatibility. This review provides a timely overview on O_2_ activation by Fe-based materials (including zero-valent iron-based materials, iron sulfides, iron (oxyhydr)oxides, and Fe-containing clay minerals) for degradation of organic pollutants. The mechanisms of O_2_ activation are systematically summarized, including the electron transfer pathways, reactive oxygen species formation, and the transformation of the materials during O_2_ activation, highlighting the effects of the coordination state of Fe atoms on the capability of the materials to activate O_2_. In addition, the key factors influencing the O_2_ activation process are analyzed, particularly the effects of organic ligands. This review deepens our understanding of the mechanisms of O_2_ activation by Fe-based materials and provides further insights into the application of this process for in situ remediation of organic-contaminated sites.

## 1. Introduction

Groundwater is a vital resource for agricultural irrigation, drinking water supply, and industrial use. However, this valuable resource is becoming increasingly more scarce due to the excessive extraction and consumption, as well as the widely occurring groundwater pollution [1,2,3,4]. Among the various sources of groundwater contamination are closed landfills without proper maintenance and “brownfield” sites, which are abandoned lands left behind after the closure or relocation of industrial or commercial facilities [5,6,7]. Soil and groundwater in a majority of the sites are contaminated with various organic pollutants [8], including organic solvents (especially chlorinated solvents) [9], petroleum hydrocarbons and gasoline products (e.g., benzene, toluene, ethylbenzene, and xylenes, collectively known as BTEX) [10], polycyclic aromatic hydrocarbons (PAHs) [11,12], pesticides [13], polybrominated diphenyl ethers [14], and perfluoroalkyl and polyfluoroalkyl substances (PFASs) [15]. For example, in approximately 80% of the Superfund sites, the groundwater is contaminated with chlorinated aliphatic hydrocarbons [16]. Due to the chemical stability, low water solubility, and propensity to adsorb onto the soil medium, these organic pollutants can persist in the subsurface for many years and pose long-term environmental risks [17,18]. These organic pollutants not only cause harm to the soil and subsurface ecological environment [19,20] but also lead to adverse effects on human health [21,22,23,24]. Numerous studies have indicated that exposure to these pollutants can lead to cancer, diabetes, respiratory and neurological diseases, and reproductive disorders [25,26,27]. For example, exposure to PAHs accounted for a significant proportion of lung cancer cases, especially in e-waste processing areas [28]. In recent years, the toxicities and health risks of emerging organic pollutants have raised increasing attention. Notably, PFASs can cause immunotoxicity, cardiotoxicity, and pancreatic and liver damage, as well as endocrine-disrupting effects [29,30]. Therefore, it is urgent to formulate effective strategies for mitigating the ecological and health risks posed by these legacy and emerging organic pollutants.

Intensive studies have been conducted to develop remediation technologies for soil and groundwater with organic contamination [31,32,33]. Among the various remediation technologies, in situ chemical oxidation (ISCO) methods have received increasing attention due to their high efficiency and simple operation [34,35,36]. During ISCO processes, oxidants such as ozone, potassium permanganate, hydrogen peroxide (H_2_O_2_), and persulfate are injected into the contaminated source zone and then activated when needed, generating stronger oxidizing species, such as hydroxyl radical (•OH) and sulfate radical [34,36,37]. However, these commonly used oxidants still suffer from some drawbacks, such as low selectivity, rapid consumption by aquifer constituents, and the risk of secondary pollution. In particular, their efficiency and cost-effectiveness for removing residual non-aqueous phase liquids (NAPL) pollutants is low.

Recent studies have found that molecular oxygen (O_2_) is an ideal green alternative to the traditional oxidants for ISCO remediation of contaminated soil/sediment and groundwater. O_2_ is relatively stable due to unfavorable one-electron reduction chemistry and spin restriction [38], which is favorable for its delivery to the pollutants without extensive consumption by aquifer constituents during its transport in the subsurface porous media. Additionally, groundwater table fluctuation, which can be caused by evaporation and rainfall, tide, lateral recharge, and drainage, results in the trapping of O_2_ in unsaturated soil and saturated aquifers [39]. Under certain conditions, O_2_ could be activated to form reactive oxygen species (ROS), such as •OH, singlet oxygen (^1^O_2_) and superoxide radical (O_2_^•−^), and H_2_O_2_ [40], mainly via electrochemical- [41], photochemical- [42], and chemical-activation approaches [38]. The electrochemical- and photochemical-activation methods require electrical power and light irradiation, as well as devices that may not be facilely emplaced underground, which greatly hinders the application of these methods for in situ remediation of contaminated sites. In contrast, the chemical activation of O_2_ by earth-abundant elements holds great promise for in situ soil and groundwater remediation.

Iron is the fourth-most-abundant element in the Earth’s crust, and Fe-containing minerals are ubiquitous in soils and aquifers [43,44,45]. Iron-based materials are extensively investigated for applications in environmental remediation, exhibiting high efficiency in degrading a range of organic pollutants (Table 1) due to the redox and catalytic activities of the Fe element and the versatility, availability, and environmental compatibility of Fe-based materials [46,47]. For example, zero-valent iron (ZVI)-based materials are the most widely used agents for in situ chemical-reduction remediation [48,49,50,51,52,53]. Meanwhile, Fe-based materials can promote ISCO remediation by the activation of H_2_O_2_/CaO_2_ [54,55,56,57,58], persulfates [59,60,61,62,63], and other oxidants [64,65,66]. Moreover, Fe-based materials such as ZVI [67,68], iron sulfides [69,70], iron (oxyhydr)oxides [71], and Fe-containing clay minerals [72] can mediate O_2_ activation to degrade organic pollutants, and the potential of Fe-mediated O_2_ activation for ISCO remediation has been actively explored in recent years. However, there is a lack of a timely review of the mechanisms and key influencing factors of O_2_ activation by iron-based materials for potential applications for in situ remediation of soil- and groundwater-suffering organic contamination.

Herein, we comprehensively review the current status of research on the activation of O_2_ by Fe-based materials, including ZVI-based materials, iron sulfides, iron (oxyhydr)oxides, and Fe-containing clay minerals for degrading contaminants commonly found in soil/sediment and groundwater. Unlike previous reviews on O_2_ activation, which highlight more efficient techniques such as electrochemical and photochemical activation for rapid abatement of pollutants (e.g., in wastewater treatment) [73,74,75,76], this review focuses on O_2_ activation by Fe-based materials without external energy input, which holds better promise for application in the remediation of organic-contaminated sites. Particularly, this review includes discussions on recent research in O_2_ activation by reduced Fe-bearing minerals abundant in soils and sediments, which has important implications for slower but sustained remediation via natural attenuation processes. The major mechanisms involved in the activation of O_2_ by the Fe-based materials are summarized, highlighting electron transfer and utilization, reaction intermediates and ROS chain reactions, and the oxidative transformation of the materials during the O_2_-activation process are discussed. Additionally, we discussed the influences of environmental and operational factors, including O_2_ concentration, organic ligands, inorganic anions, and microbial activity, on the O_2_ activation and pollutant-degradation performance by iron-based materials. This review also identifies limitations of current studies and suggests future research directions to enhance understanding of O_2_ activation by iron-based materials and its applications in soil and groundwater remediation.

## 2. Activation of O_2_ by Fe-Based Materials

### 2.1. ZVI-Based Materials

**Figure 1:**
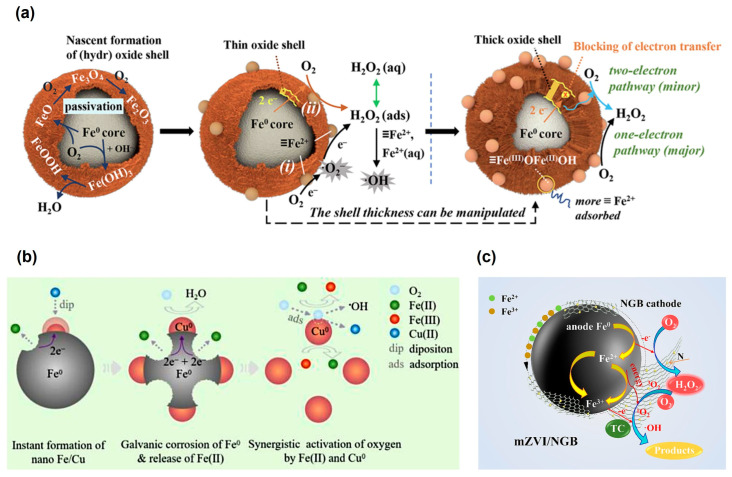
(**a**) Illustration of the mechanisms of O_2_ activation by Fe^0^ with an oxide shell of different thickness [73]. (**b**) Illustration of the mechanisms of Cu-enhanced •OH production in Cu^0^/ZVI system under oxic condition [93]. (**c**) Illustration of the catalytic mechanism of O_2_ by mZVI/N-doped graphene-like biochar (mZVI/NGB) [97].

Materials containing ZVI have been commonly employed as remediation agents for reductive degradation of organic pollutants under anaerobic conditions due to the high reducing capacity of elemental Fe (Table 2) [77,78,79,80,81,82,83]. However, studies have indicated that the degradation efficiency of organic pollutants by ZVI is significantly higher in O_2_-containing aqueous solutions than under anaerobic conditions [67,84,85], and O_2_ activation by ZVI-based materials has recently been extensively explored for degrading various organic pollutants (Table 3). This increased efficiency is primarily attributed to the reaction between ZVI and O_2_, which leads to the generation of ROS [67,68]. The mechanism of ZVI-mediated O_2_ activation for the generation of ROS involves two-electron transfers from Fe^0^ to adsorbed O_2_, producing Fe(II) and H_2_O_2_. And the release of Fe(II) further induces O_2_ activation via a sequential single-electron transfer process, generating •OH, O_2_^•−^, and other ROS (Equations (1)–(6)) [86]. The yield of •OH is primarily affected by the reaction between Fe^0^ and O_2_ via four-electron transfer without ROS generation (Equation (7)) [87]. Due to this reaction pathway, only less than 10% of the ZVI is utilized for contaminant transformation under oxic conditions [88]. Therefore, the yield of ROS decreases with increasing pH due to the inhibition of Fe(II) release via the four-electron-transfer reaction under high pH conditions [84]. Notably, the ROS generation is influenced by the oxide layer on the surface of ZVI, in a fashion dependent on the thickness of the layer [89]. The iron oxide layer can adsorb ferrous ions, which can activate O_2_ through a single-electron-transfer pathway. When the iron oxide layer is thin, both the Fe^0^ core-mediated two-electron transfer and the surface-bound/adsorbed Fe(II)-mediated single-electron transfer play significant roles in O_2_ activation [73]. However, as the thickness of the oxide layer increases, it inhibits electron transfers from the Fe^0^ core to adsorbed O_2_. Meanwhile, more ferrous ions are adsorbed on the surface, and these surface-bound Fe(II) become the primary species responsible for O_2_ activation (Figure 1a) [75,89,90].

Fe^0^ + O_2_ + 2H^+^ → Fe(II) + H_2_O_2_ (1)

Fe(II) + O_2_ → Fe(III) + O_2_^•−^ (2)

Fe(II) + O_2_^•−^ + 2H^+^ → Fe(III) + H_2_O_2_ (3)

O_2_^•−^ + H^+^ → HO_2_^•^ (4)

HO_2_^•^ + HO_2_^•^ → H_2_O_2_ + O_2_ (5)

Fe(II) + H_2_O_2_ → Fe(III) + •OH + OH^−^ or Fe(IV) + 2OH^−^ (6)

2Fe^0^ + O_2_ + 4H^+^ → 2Fe(II) + 2H_2_O (7)

To improve the O_2_-activation efficiency and ROS yield, various modified ZVI materials have been developed, notably by doping with metal and non-metal elements or immobilizing ZVI on porous materials. Inspired by the galvanic corrosion between connected dissimilar metals [91], a series of ZVI-based bimetallic materials were designed. In a Cu^0^/ZVI material, Cu^0^ can enhance the reaction potential by forming infinite galvanic cells with Fe^0^, thereby significantly accelerating Fe-mediated O_2_ activation [92]. Additionally, Cu accelerates the release of Fe(II) species during O_2_ activation (Figure 1b). Furthermore, Cu species can also effectively facilitate the iron cycle and serve as new active sites (Equations (8)–(12)) [93]. However, it has also been suggested that Cu may inhibit •OH generation due to the formation of a passivation layer of Cu oxides [104]. This discrepancy may be attributed to the complex chain reactions and the dosage of Cu, which should earn more attention in further studies. Another metal element, Ni, has been shown to reduce the proportion of the four-electron-reaction pathway of ZVI by increasing Fe(II) release [88]. Alternatively, the Fe–Mg bimetallic material can increase the degradation of 4-chlorophenol by enhancing the generation of surface-bound •OH [94]. Recently, a Fe–Mn core-shell bimetallic material was reported for O_2_ activation and on-site generation of H_2_O_2_, and it was proposed that the amorphous Mn shell can not only protect the Fe core from excessive oxidation, thereby increasing electron utilization, but also contain abundant structural defects, which serve as efficient catalytic sites [95]. Doping with non-metal elements can also affect the efficiency of O_2_ activation by ZVI materials. For example, the incorporation of chloride ions into microscale zero-valent iron (mZVI) can create oxygen vacancies (OVs), resulting in abundant adsorbed ferrous ions and accelerated electron transfer [105]. Sulfidation is one of the most effective methods to improve the efficiency and selectivity of reductive degradation of pollutants by nanoscale ZVI (nZVI) [106,107,108,109], and it has been demonstrated that the presence of S could enhance electron transfers from the Fe^0^ core to surface Fe(III) and O_2_ via the single-electron pathway, thus promoting pollutant degradation under aerobic conditions [96,110].

Cu^0^ + Fe(III) → Cu(I) + Fe(II) (8)

Cu(I) + Fe(III) → Cu(II) + Fe(II)(9)

Cu^0^ + O_2_ + 2H^+^ → Cu(II) + H_2_O_2_ (10)

Cu(I) + O_2_ → Cu(II) + O_2_^•−^ (11)

Cu(I) + O_2_^•−^ + 2H^+^ → Cu(II) + H_2_O_2_ (12)

Another approach to enhancing ZVI performance involves immobilizing ZVI on porous supports, particularly carbon materials. The carbon materials (e.g., graphene), usually with a conjugated network skeleton of sp^2^ hybridized carbon atoms, could enhance electron transfer and modify the adsorption/dissociation energy of O_2_, making O_2_ activation thermodynamically and kinetically more favorable [111,112,113]. Meanwhile, the combination of ZVI with graphene (3D-GN@nZVI) can also greatly inhibit the reduction of O_2_ to H_2_O via a four-electron process, thereby increasing the yield of ROS [98]. Recent studies have indicated that a mZVI/N-doped graphene-like biochar composite (mZVI/NGB) could promote the contribution of non-radical pathways (^1^O_2_ and electron transfer) during O_2_ activation for antibiotics degradation (Figure 1c) [97]. Similarly, for Fe/Cu-biochar (Cu/Fe-BC) materials, both radicals (e.g., O_2_^•−^, •OH) and non-radicals (e.g., ^1^O_2_) were detected as the dominant reactive species, enabling the efficient degradation of a wide spectrum of organic pollutants, even in the presence of various interfering substances [99]. In contrast, O_2_^•−^ and H_2_O_2_ are the key ROS in the ZVI–biochar system, indicating that the secondary metal has a significant influence on the O_2_-activation pathway [100]. Carbon nanotubes (CNTs) have also been demonstrated as an excellent support material to regulate ROS generation dynamics by bimetallic Fe-based materials. For example, in a Zn–Fe–CNT composite, CNTs effectively collect electrons from Zn^0^ nanoparticles and reduce O_2_ to H_2_O_2_, which was subsequently converted to •OH by Fe^0^ nanoparticles [101,102]. Due to the synergistic effects, the composite achieved excellent performance for the degradation of 4-chlorophenol and sulfamethoxazole (Table 3). In addition to carbon materials, nZVI can also be successfully incorporated within the channels of monodisperse mesoporous silica nanospheres (nZVI@MSN) to increase its stability and durability [103].

### 2.2. Iron Sulfides

#### 2.2.1. Pyrite

Pyrite (FeS_2_) is the most widely distributed stable-phase iron sulfide mineral in Earth’s crust [114]. The oxidation of natural pyrite, which can lead to the generation of H_2_O_2_, has been confirmed under anaerobic conditions [115]. The mechanism involved in this process is primarily attributed to the presence of surface defects, arising from the cleavage of the S–S bond [116]. As a result, transient S– and ≡Fe(III) dangling bonds are generated at these sulfur-deficient defect sites, which can induce the formation of •OH by extracting an electron from adsorbed H_2_O, and the •OH radicals then combine to form H_2_O_2_ in the absence of O_2_ [117]. In a recent study investigating oxidation of benzoic acid by sulfur vacancy (SV)-rich FeS_2_ in isotopically labeled H_2_^18^O, the generation of ^18^O-containing *p*-hydroxybenzoic acid was observed, which further provided direct evidence that the anaerobic oxidation of water by SV-rich FeS_2_ is responsible for the generation of •OH at the pyrite–water interface [118]. However, it was noted that the •OH generated through this mechanism was insufficient to achieve an obvious degradation of organic pollutants. In contrast, efficient pollutant removal can be achieved in pyrite suspension with sufficient O_2_ (Table 4), which highlights the crucial role of pyrite oxidation by O_2_ [70,119].

**Table 4:** Degradation of organic pollutants via O_2_ activation by iron sulfides.

Material	Pollutant	Removal Ratio (%)	Reaction Time (h)	pH	Rate Constant	Reference
Pyrite	Trichloroethylene	100	323	4.0	0.013 h^−1^	[70]
Pyrite	Acid orange 7	52.8	5	6.3	N/A	[120]
Pyrite	Carbamazepine	81.5	24	7.0	0.103 ± 0.001 h^−1^	[121]
Pyrite	Phenol	76.8	24	7.0	0.084 ± 0.001 h^−1^	[121]
Pyrite	Bisphenol A	100	24	7.0	0.147 ± 0.001 h^−1^	[121]
SV-rich pyrite	Sulfamethoxazole	88.3	12	8.5	29.2 × 10^−4^ h^−1^	[122]
Pyrite	Sulfamethoxazole	70.0	12	4.0	0.095 h^−1^	[123]
Mackinawite	Flumequine	79.8	4	7.0	51.6 × 10^−3^ min^−1^	[124]
Mackinawite	Enrofloxacin	87.7	4	7.0	34.0 × 10^−3^ min^−1^	[124]
Mackinawite	Ciprofloxacin	81.5	4	7.0	25.4 × 10^−3^ min^−1^	[124]
Mackinawite	Trichloroethylene	23.4 *	3	7.0	2.07 × 10^−3^ min^−1^	[69]
Mackinawite	Phenol	34.1 *	3	7.0	3.53 × 10^−3^ min^−1^	[69]
Surface-oxidized mackinawite	Phenol	17.5 *	1.5	7.3	3.8 × 10^−3^ min^−1^	[125]

Note: N/A, data not available. SV, sulfur vacancy. *, data obtained from the literature using the Getdata 2.26 software.

**Figure 2:**
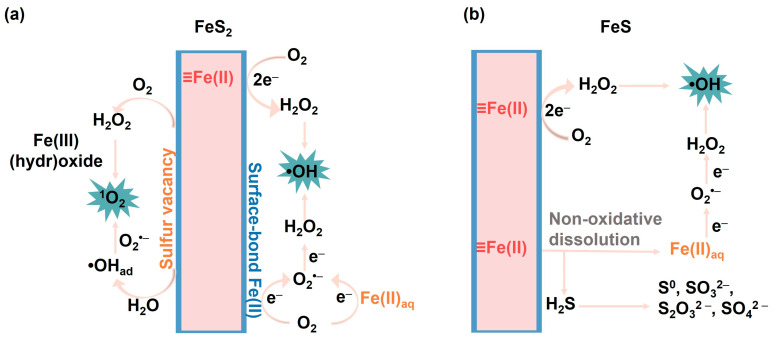
Illustration of the mechanisms of O_2_ activation by (**a**) FeS_2_ and (**b**) FeS.

The mechanism for O_2_ activation by pyrite can be understood from an analysis of the Fe species in pyrite suspension (Equations (13)–(18)). Previous studies have proposed that structural Fe(II) and surface-bound Fe(II) can mediate either a two-electron-transfer pathway or two separate one-electron-transfer processes, with H_2_O_2_ or O_2_^•−^ as intermediates, ultimately leading to the formation of •OH (Figure 2a) [119,126]. Another pathway involves the leaching of dissolved Fe(II) from bulk FeS_2_, which mediates O_2_ activation in aqueous solution via a single-electron-transfer pathway [127]. However, the contribution of this approach is generally minor due to the stable structure and extremely low dissolution rate of pyrite even under acidic conditions [120,128]. Moreover, the reactivity of dissolved Fe(II) is lower than the structural Fe(II) and surface-bound Fe(II) according to their redox potentials (Table 2) [72,129]. Note that, although S_2_^2−^ has the capability to reduce surface-bound Fe(III), it does not directly participate in O_2_ reduction [130]. This inference is substantiated by the results of in situ horizontal attenuated total reflectance infrared spectroscopy and isotope analysis of reaction products (e.g., SO_4_^2−^ and iron oxyhydroxide), which demonstrated that the O atoms in SO_4_^2−^ primarily originate from H_2_O, while the O atoms in the iron oxyhydroxide are derived from O_2_ [131,132].

≡Fe(II) + O_2_ + 2H^+^ → Fe(III) + H_2_O_2_ (13)

Fe(II)_ad_ + O_2_ → Fe(III) + O_2_^•−^ (14)

Fe(II) + O_2_^•−^ + 2H^+^ → Fe(III) + H_2_O_2_ (15)

Fe(II)_(aq)_ + O_2_ → Fe(III)_(aq)_ + O_2_^•−^ (16)

Fe(II)_(aq)_ + O_2_^•−^ + 2H^+^ → Fe(III)_(aq)_ + H_2_O_2_ (17)

H_2_O_2_ + Fe(II) → Fe(III) + •OH + OH^−^ (18)

As an interfacial reaction, the efficiency of O_2_ activation by pyrite is dictated by the surface properties of pyrite, which in turn is dependent on the exposed facets, the presence of surface defects, and the formation of the iron (oxyhydr)oxide layer. The exposed facets of pyrite significantly influence the O_2_-activation configurations and electron-transfer ability [133]. Notably, it has been recently revealed that pyrite crystals with more exposed {210} facets exhibit higher generation rates of •OH and other ROS (e.g., O_2_^•−^ and H_2_O_2_) due to different surface electron-donating capacities and kinetics among different facets. Correspondingly, facet-dependent degradation of organic pollutants (e.g., carbamazepine, phenol, and bisphenol A) was achieved [121]. Another recent study highlighted that SV sites in pyrite can activate O_2_ via a two-electron-transfer mechanism, generating ^1^O_2_, which played a key role in the selective degradation of sulfamethoxazole [122]. The generation of ^1^O_2_ arises from the breakage of the O–H bond in H_2_O_2_, facilitated by Fe(III) (oxyhydr)oxide on the pyrite surface, as presented by Equations (19) and (20) [122], whereas another recent study proposed that ^1^O_2_ could also form through the interaction between surface-bound •OH and O_2_^•−^ in the pyrite-oxidation process (Equations (21) and (22)) [123]. The iron (oxyhydr)oxides on the surface of pyrite, which quickly forms after pyrite is exposed to O_2_, can also affect ROS generation during O_2_ activation in the pyrite system in both positive and negative ways. The formation of iron (oxyhydr)oxides can provide fast electron-transfer channels by establishing a potential gradient between the two mineral phases [134]. Additionally, the iron (oxyhydr)oxides can adsorb more surface-bound Fe(II) by forming inner-sphere complexes with surface groups, thereby enhancing O_2_-reduction efficiency [135]. However, other studies indicated that the (oxyhydr)oxide coating tends to lower the H_2_O_2_ utilization by catalyzing its transformation into H_2_O, resulting in the decrease of ROS concentration in the system [136,137,138].

Fe(III)_(SV-Pyrite)_ + O_2_ → H_2_O_2_ (19)

H_2_O_2_ → ^1^O_2_ + 2H^+^ (20)

Fe(III)_(SV-Pyrite)_ + H_2_O → Fe(II)_(SV-Pyrite)_ + •OH_ad_ + H^+^ (21)

O_2_^•−^ + •OH_ad_ → ^1^O_2_ + OH^−^ (22)

#### 2.2.2. Mackinawite

Mackinawite (FeS) is a metastable iron sulfide mineral, which can readily convert into more stable phases, such as FeS_2_ and greigite (Fe_3_S_4_) in a natural environment [139]. Due to its structural instability and reducing power, FeS is prone to oxidation by O_2_, and oxidative transformation of pollutants (e.g., As(III) and U(IV)) has been observed during FeS oxidation under aerobic conditions, and different oxidative species (e.g., •OH, Fe(IV), and transient surface Fe(III) species) have been proposed to initiate the pollutant degradation [140,141,142]. Yuan’s group confirmed the production of •OH during the oxidation of FeS by O_2_ and demonstrated that the produced •OH played a key role in the oxidation of As(III) [143]. The rate of •OH formation by mackinawite is one-to-two orders of magnitude higher than that observed for other forms of reduced iron minerals, such as nontronite, pyrite, and siderite (FeCO_3_), under comparable conditions due to the metastable structure and higher Fe(II) content [143,144]. The ROS produced during FeS oxidation can also efficiently degrade various organic pollutants, such as phenol, trichloroethylene (TCE), and fluoroquinolone antibiotics [69,124].

The mechanism of FeS oxidation by O_2_ is dependent on the pH conditions. When pH is lower than 3, FeS primarily undergoes non-oxidative dissolution, and most of the structural Fe(II) in FeS enters the aqueous solution before undergoing oxidation, which leads to an increase in dissolved ferrous ion concentration and the generation of H_2_S (Equation (23)) (Figure 2b) [140,145]. The dissolved ferrous ions can mediate homogeneous Fenton processes [127]. Surface-mediated oxidative dissolution also occurs under acidic conditions, due to the formation of an iron (oxyhydr)oxide layer, which can adsorb Fe(II) on the surface, activating O_2_ to form ROS [135]. Under neutral pH conditions, due to the low concentration of dissolved iron ions, surface-mediated oxidation mechanisms predominate, with surface species of FeS transforming from ≡Fe(II)–S through ≡Fe(III)–S to ≡Fe(III)–O in the presence of O_2_ [140]. This structural Fe(II)-mediated heterogeneous reaction dominates the O_2_ activation and ROS production reactions in the FeS system under neutral conditions [143], which involves a two-electron-transfer mechanism, leading to the generation of H_2_O_2_ intermediate and subsequent formation of •OH [143,146]. Notably, in addition to aqueous •OH, other active species such as high-valent iron, surface-bound •OH, or sulfur-based radicals may also be present in the FeS/O_2_ system [147]. Apart from structural Fe(II), S(–II) can also act as the electron donator, mediating the iron cycle without directly participating in the O_2_-activation process [143]. As with FeS_2_, the Fe (oxyhydr)oxide coatings formed on the surface of FeS could affect O_2_-activation efficiency by mediating electron transfers from FeS to O_2_. Interestingly, the storage of partially oxidized FeS under anoxic conditions could change its mineralogical structure and surface Fe speciation, forming new Fe(II) species in the (oxyhydr)oxide layer, which leads to enhanced reactivity toward O_2_ and the production of ROS [125].

FeS + 2H^+^ → Fe(II)_(aq)_ + H_2_S_(aq)_ (23)

### 2.3. Iron (Oxyhydr)Oxides

Magnetite (Fe_3_O_4_) is one of the most widely distributed reductive iron oxides in the subsurface environment, and it can activate O_2_ to generate O_2_^•−^, H_2_O_2_, and •OH and has been used for organic pollutant degradation (Table 5) [71,148,149]. Structural Fe(II) is considered the dominant species participating in O_2_ activation in magnetite via a single-electron transfer under alkaline conditions, whereas dissolved iron ions originating from the dissolution of magnetite can participate in O_2_ activation at pH < 6.5 [71]. Interior structural iron could facilitate O_2_ activation by transferring electrons to surface iron and accelerating the iron cycle [150]. Nevertheless, studies have indicated that only half of the total Fe(II) in magnetite could be effectively utilized due to the low inner-electron-transfer ability, leading to a low O_2_-activation efficiency [71]. Notably, it has been proposed that the presence of OVs can change the Gibbs free energy for the generation of adsorbed O_2_ intermediate, making the reduction of O_2_ thermodynamically more favorable and facilitating the electron transfer [148].

To improve the efficiency of O_2_ activation, many synthetic Fe_3_O_4_-based composite materials have also been designed. For example, in a carboxylated Cu^0^/Fe_3_O_4_ system, Cu^0^ can act as the reducing agent and accelerate the regeneration of surface iron. In addition, Cu^0^ serves as a new O_2_ activation site to further increase the generation of H_2_O_2_ in the system via two-electron transfer, leading to the efficient removal of chlorophenol [151]. Similarly, Fe_3_O_4_ can mediate the ROS-generation dynamics of carbon-supported zero-valent metal, thus increasing the overall O_2_-activation efficiency. For example, in a Zn^0^–CNT–Fe_3_O_4_ composite, with Zn^0^ and Fe_3_O_4_ nanoparticles well dispersed on the surface of CNTs, the self-decomposition of H_2_O_2_ (generated from O_2_ reduction by Zn^0^ on CNTs surface) into H_2_O is significantly inhibited. Meanwhile, the conversion of H_2_O_2_ into •OH rapidly occurs with a high yield [152]. As a result, the Zn^0^–CNT–Fe_3_O_4_ composite exhibited approximately four times higher removal efficiency for 4-chlorophenol than that by CNT-Fe_3_O_4_ and Zn^0^–CNT materials [152]. Moreover, sulfidation can lead to higher H_2_O_2_ and •OH production during Fe_3_O_4_ oxidation because surface sulfur species can decrease electron-transfer resistances of Fe_3_O_4_, thereby accelerating the electron transfer from interior structural iron to the surface Fe(III) and facilitating the reaction between surface iron and O_2_ [155]. Moreover, for a vacancy-rich iron-cobalt bimetallic composite prepared by ball milling CoS_2_ and Fe_3_O_4_ (b-CoS_2_/Fe_3_O_4_), the interfacial interaction between CoS_2_ and Fe_3_O_4_ can change the Fe–O bond energy of Fe_3_O_4_, thereby accelerating the formation of surface-bound Fe(II), which in turn promotes O_2_ activation by the composite [153].

Fe(III) (oxyhydr)oxides such as goethite (α-FeOOH) and hematite (α-Fe_2_O_3_) generally lack the ability to reduce O_2_ due to the +3 valence state of iron in these compounds. However, studies have indicated that Fe(III) (oxyhydr)oxides can adsorb Fe(II) on their surface, and these surface-bound Fe(II) can activate O_2_ through a single-electron-transfer process [156,157]. Furthermore, the incorporation of Cu into goethite can increase the O_2_-activation ability of surface-bound Fe(II) by modifying the adsorption energy of O_2_, lowering its oxidation potential, and increasing the interfacial electron-transfer process on Fe(III) (oxyhydr)oxides [156]. The incorporation of secondary metal atoms can also increase the content of OVs in hematite, further enhancing the electron-transfer efficiency [150]. Notably, the incorporation of secondary metals does not necessarily have a positive effect on the O_2_-activation efficiency. For example, the incorporation of Zn results in a higher oxidation potential for Fe(II) oxidation, which is unfavorable for the O_2_-activation process [150]. Moreover, it was recently proposed that Fe(III) (oxyhydr)oxides can serve as an electron-transfer mediator for O_2_ reduction by reducing organic compounds to generate ROS. Specifically, Fe(III) on their surface receives electrons from the reducing organic compounds, such as thiols, to form surface Fe(II), which then mediate the activation of O_2_ efficiently [154].

### 2.4. Fe(II)-Containing Clay Minerals

**Figure 3:**
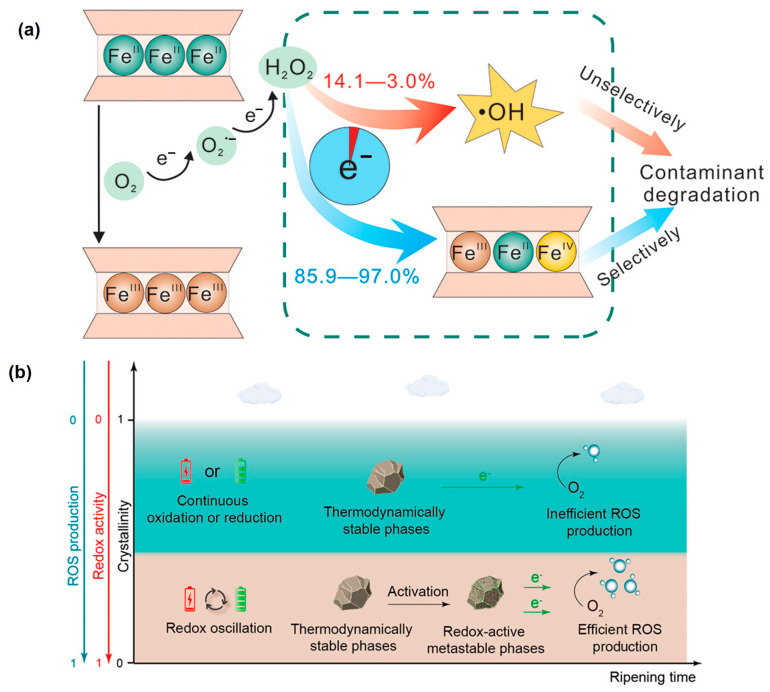
(**a**) Illustration of edge surface Fe(II) in clay mineral favoring the Fe(IV) generation over •OH generation [165]. (**b**) Illustration of redox oscillations activating thermodynamically stable iron minerals for enhanced ROS production [167].

Fe(II)-containing clay minerals are widely present in subsurface environments, such as sediments and soils [158]. Recent studies have shown that the oxidation of Fe(II)-containing clay minerals, such as smectites [72] (particularly reduced nontronite [159,160]) and illite [161], is one of the important sources of environmental radicals, which deeply affects the attenuation behavior of pollutants, including 1,4-dioxane, TCE, phenol, and PAHs (Table 6). The iron contents in these clay minerals, which range from about  2 wt.% in montmorillonite to about 30 wt.% in nontronite [162], is a crucial factor influencing the ROS yield [72]. In addition to total Fe content, the different forms of Fe species in Fe-containing clay minerals, including structural Fe(II), surface-bound Fe(II), and exchangeable Fe(II), also have a significant impact on the generation of ROS. Structural Fe(II) is generally the dominant species for O_2_ activation by Fe-containing clay minerals [163,164]. The reactivity of structural Fe(II) is highly affected by its coordination environment. Specifically, structural Fe(II) at the edge (Fe(II)_edge_) is coordinated by electron-rich ligands (e.g., ≡O–, ≡HO–, and ≡Fe(II)–O–), which exhibit high activity for O_2_ activation and preferentially lead to the generation of Fe(IV), along with a low •OH yield (Figure 3a). In contrast, interior Fe(II) (Fe(II)_int_) tends to be coordinated by electron-poor ligands (e.g., ≡Al(III)–O– and ≡Fe(III)–O–). Although Fe(II)_int_ is much less active than Fe(II)_edge_, it can selectively activate O_2_ to •OH [165]. Additionally, the Fe(III)_edge_ can be regenerated through various pathways, such as the continuous supply of electrons from interior adjacent Fe(II) via single-electron transfer until the electrons in the interior Fe(II) are eventually depleted [72,166].

**Table 6:** Degradation of organic pollutants via O_2_ activation by Fe(II)-containing clay minerals and soils/sediments.

Material	Pollutant	Removal Ratio (%)	Reaction Time (h)	pH	Rate Constant	Reference
Nontronite	1,4-Dioxane	78.8 *	120	7.0	N/A	[161]
Illite	1,4-Dioxane	34.3 *	120	7.0	N/A	[161]
Montmorillonite	1,4-Dioxane	27.4 *	120	7.0	N/A	[161]
Reduced nontronite	Trichloroethylene	50.0	0.5	7.5	N/A	[160]
Riparian sediment	Trichloroethylene	27.6	6	7.0	N/A	[164]
Lakeshore sediment	Trichloroethylene	19.1	6	7.0	N/A	[164]
Pond sediment	Trichloroethylene	15.4	6	7.0	N/A	[164]
Nontronite	Phenol	43.1	6	7.0	N/A	[165]
Montmorillonite	Phenol	59.8	6	7.3	N/A	[165]
Sandbeach sediment	Phenol	9.95 *	10	6.96	N/A	[168]
Lakeshore sediment	Phenol	39.3 *	10	7.15	N/A	[168]
Farmland sediment	Phenol	48.5 *	10	Unadjusted	N/A	[168]
Paddy soils	Naphthalene	76.0 *	252	Unadjusted	N/A	[169]
Paddy soils	Phenanthrene	49.6 *	252	Unadjusted	N/A	[169]
Paddy soils	Pyrene	28.6 *	252	Unadjusted	N/A	[169]

Note: N/A, data not available. *, data obtained from the literature using the Getdata 2.26 software.

In addition to structural Fe(II), surface-bound Fe(II) also plays an important role in ROS generation and pollutant degradation by Fe(II)-containing clay minerals [168]. Similar to structural Fe(II), the reactivity of surface-bound Fe(II) is also highly dependent on its coordination environment. Specifically, the sequence of surface-bound Fe(II) reactivity probably follows the order of ≡Si(IV)–O–Fe(II) < ≡Al(III)–O–Fe(II) < ≡Fe(III)–O–Fe(II) < ≡Fe(II)–O–Fe(II) < ≡HO–Fe(II) [169]. Additionally, the coordination environment of surface-bound Fe(II) also affects the O_2_-activation mechanism. When surface-bound Fe(II) is coordinated with electron-rich ligands, it can efficiently activate O_2_. Nonetheless, due to the inner-sphere complexation with O_2_, more non-•OH species (e.g., Fe(IV)) are generated. Conversely, when coordinated with electron-poor ligands, surface-bound Fe(II) exhibits a low reactivity toward O_2_, but more •OH is generated, via an outer-sphere interaction mechanism with O_2_ [168,169]. Compared to structural Fe(II) and surface-bound Fe(II), exchangeable Fe(II) contributes minimally to •OH formation and can even exhibit a scavenging effect against •OH [169].

In addition to engineered Fe-based materials and isolated Fe-bearing minerals, recent studies have increasingly focused on the generation of ROS in actual soils and sediments under the redox-fluctuation condition, and Fe(II)-bearing compounds play key roles in this process [164,170,171,172,173,174,175]. The soil–water interface was generally considered as the active zone for intense H_2_O_2_ and •OH production due to the limited oxygen penetration and the rapid turnover of the reducing and oxidizing substances at the redox interfaces [170,171]. Although the yield of ROS varies across different sediments due to their unique physicochemical properties, for specific sediment, surface-bound Fe(II) and structural Fe(II) in poorly crystalline iron minerals are the primary contributors to ROS production [168,176]. Model studies indicate that the relative contributions of surface-adsorbed Fe(II) and structural Fe(II) in •OH production are 16.4–33.9% and 66.1–83.6% in sediment, respectively [164]. It has recently been proposed that tidal hydrology-triggered redox fluctuation could promote ROS generation by accelerating the production of reactive ferrous ions and amorphous ferric oxyhydroxides, thereby promoting surface electrochemical activities and O_2_-activation capability (Figure 3b) [167]. These results further confirm the vital role of natural iron minerals in ROS generation under dark conditions.

## 3. Influencing Factors

The efficiency of O_2_ activation by iron-based materials is influenced by several key environmental or operational factors, including O_2_ concentration, organic ligands, inorganic anions, and microbial activity. Moreover, some factors can change the O_2_-activation mechanism and pathways. This section summarizes the effects of these factors on the efficiency and mechanisms of O_2_ activation by iron-based materials.

### 3.1. O_2_ Concentration

With O_2_ being the precursor to the generated ROS, increasing the O_2_ concentration commonly results in a higher ROS concentration during O_2_ activation by Fe-based materials [69,70,72]. However, it has been reported that, for Fe-containing clay minerals, excessively high levels of O_2_ would lead to adverse effects on the generation of ROS due to the ineffective oxidation of structural Fe(II) [177]. Additionally, in systems where oxidation and reduction transformation processes of pollutants occur simultaneously, the concentration of O_2_ also influences the reaction pathway and products of organic pollutants. For example, in the aerobic degradation of TCE by ferrous minerals in natural sediments, more low-molecular-weight acids were generated when O_2_ concentration exceeded 120 μM, while only acetylene and/or ethene were observed when O_2_ concentration was lower than 26 μM [178]. Furthermore, O_2_ concentration also affects the transformation behaviors of the iron-bearing minerals. For example, high O_2_ concentration promotes the dissolution of FeS and facilitates the formation of reactive iron hydroxides/oxides, such as lepidocrocite, while Fe_3_S_4_ is generated in the absence of O_2_ [144]. These O_2_-concentration-dependent transformation products exhibit different capabilities in generating ROS such as •OH [144].

### 3.2. Organic Ligands

**Figure 4:**
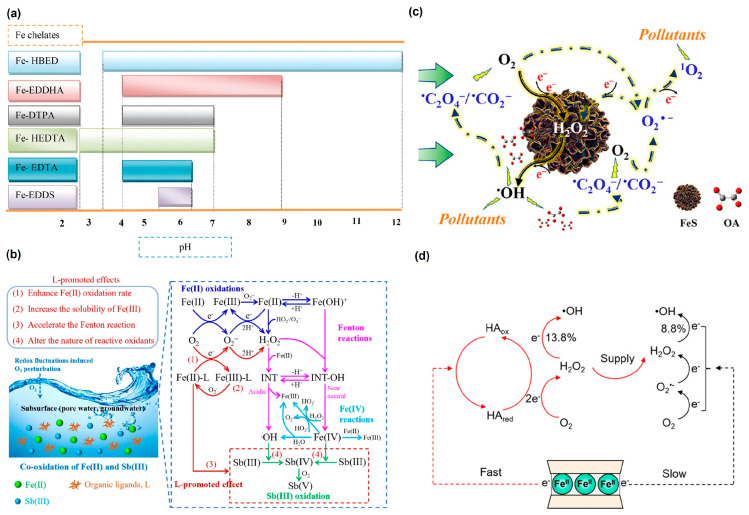
(**a**) Summary of pH ranges over which Fe chelates are stable [179]. (**b**) Reaction mechanisms of ligand-enhanced Fe(II) oxidation [183]. (**c**) Illustration of OA-enhanced FeS oxygenation mechanism [190]. (**d**) Illustration of the mechanisms for HA-enhanced oxygenation of Fe-containing clay mineral [191].

Both natural and synthetic organic ligands exist in the subsurface environment and significantly affect the efficiency of Fe-based materials in mediating O_2_ activation. In general, synthetic organic ligands have greater influences on O_2_ activation than natural ligands. Synthetic organic ligands, such as ethylenediaminetetraacetic acid (EDTA) and N,N′-1,2-ethanediylbis-1-aspartic acid (NTA), are well known for their outstanding ability to form stable complexes with iron ions across a wide pH range (Figure 4a) [179], thereby influencing Fe(II) oxidation kinetics and the iron cycle via modifying the redox potential [180,181]. Furthermore, these ligands have been found to promote the release of active iron species from the bulk materials through surface-polarization reactions [182] or the proton-coupled electron-transfer process (Figure 4b) [183,184,185]. The release of Fe into the aqueous solution depends on the concentration and complexing ability of ligands (Table 7) [83,186,187]. Specifically, EDTA, which has a particularly strong complexation ability, can form monodentate inner-sphere Fe(II)-EDTA complexes on the surface ZVI. When the concentration of EDTA is significantly lower than that of ZVI, only low levels of dissolved Fe(II) are detected in the solution. In this case, a heterogeneous reaction dominated by Fe(II)_ad–_EDTA complexes is responsible for O_2_ activation and ROS generation [188]. However, when the dosages of EDTA and ZVI are on the same order of magnitude, the Fe(II)_aq–_EDTA complex in the solution predominantly drives the reactions, and free •OH in the solution is primarily responsible for removing organic pollutants [184]. Additionally, the configurations of the Fe–ligand complexes also significantly affect the efficiency of O_2_ activation. For example, the Fe(II)–NTA complex is more efficient in activating O_2_ than the Fe(II)–EDTA complex due to the relatively open structure, leading to more ROS generation under the same condition [188]. Moreover, some organic ligands with reducing capabilities, such as hydroxylamine [189], can even directly reduce Fe(III) to Fe(II), resulting in a more efficient Fe cycle and higher ROS yield. Notably, the quenching effect of the ligands on •OH (Table 7) should be also considered for a more accurate analysis of the contributions of ROS to pollutant degradation kinetics.

Compared to synthetic ligands, natural organic acids, including citric acid (CA), oxalate (OA), and humic acid (HA), generally exhibit a relatively weak ability to form complexes with iron. However, they can still modify the redox potential of iron ions and accelerate the iron cycle and O_2_ activation effectively [192]. Moreover, reductive natural organic acids, such as glutathione [193], ascorbic acid [194], and protocatechuic acid [195], have the potential to reduce Fe(III) to Fe(II) directly. Studies have indicated that the dissolved Fe(II)–ligand complexes can mediate one electron-transfer process and play a dominant role in O_2_ activation by FeS in the presence of CA and OA [192]. However, the capability of these organic acids to promote the dissolution of FeS_2_ is lower than for FeS due to the more stable structure of FeS_2_. Correspondingly, dissolved Fe(II)-mediated O_2_ activation is less important in FeS_2_ suspension [182,192]. These results indicated that the effect of organic acids on the contribution of homogeneous reactions to overall O_2_ activation efficiency is closely related to the properties of the materials. Notably, it was recently revealed that organic ligands can promote ROS generation during the oxygenation of FeS minerals by producing abundant carbon-centered radicals. For example, OA could be preferentially oxidized by •OH, leading to the generation of carbon-centered radicals (e.g., •C_2_O_4_^−^ and •CO_2_^−^), which further supply electrons to O_2_ and contribute to at least 93.6% of the total •OH production in the FeS/OA/O_2_ system (Figure 4c) [190].

As an important constituent of natural organic matter, HA has been confirmed to promote the generation of •OH by forming an aqueous Fe–HA complex and promoting the regeneration of Fe(II) via its reduced functional groups [196,197,198,199]. Meanwhile, microbially or chemically reduced HA has the potential to directly activate O_2_ via the active quinone groups (−137 to −225 mV vs. NHE) to generate ROS [200]. Moreover, HA can mediate heterogeneous O_2_ activation by Fe-containing minerals. It has been recently proposed that the presence of HA could change the reaction mechanism of nontronite oxygenation, where HA accepts electrons from the structural Fe and then delivers the elections to O_2_ through two-electron-transfer pathways (Figure 4d) [191]. Compared to the direct electron transfer from structural Fe to O_2_, reduced HA exhibits a faster O_2_-reduction rate and higher selectivity for •OH. The HA-mediated pathways contributed to 70% of H_2_O_2_ and 62.1% of •OH generation in the HA/nontronite system. However, other studies have shown that the presence of HA could slow down the oxidation of reduced Fe-bearing clay minerals due to the competitive adsorption with O_2_ [201].

Organic ligands can also affect the generation of non-hydroxyl radical species during the Fe-based material-mediated O_2_-activation process. For example, the presence of CA could promote the generation of ^1^O_2_ in the S–nZVI/O_2_ systems because the Fe(II)–CA complex promotes the generation of more O_2_^•−^, which could further react with H_2_O/H^+^ to generate ^1^O_2_ and H_2_O_2_ [202].

### 3.3. Inorganic Anions

**Figure 5:**
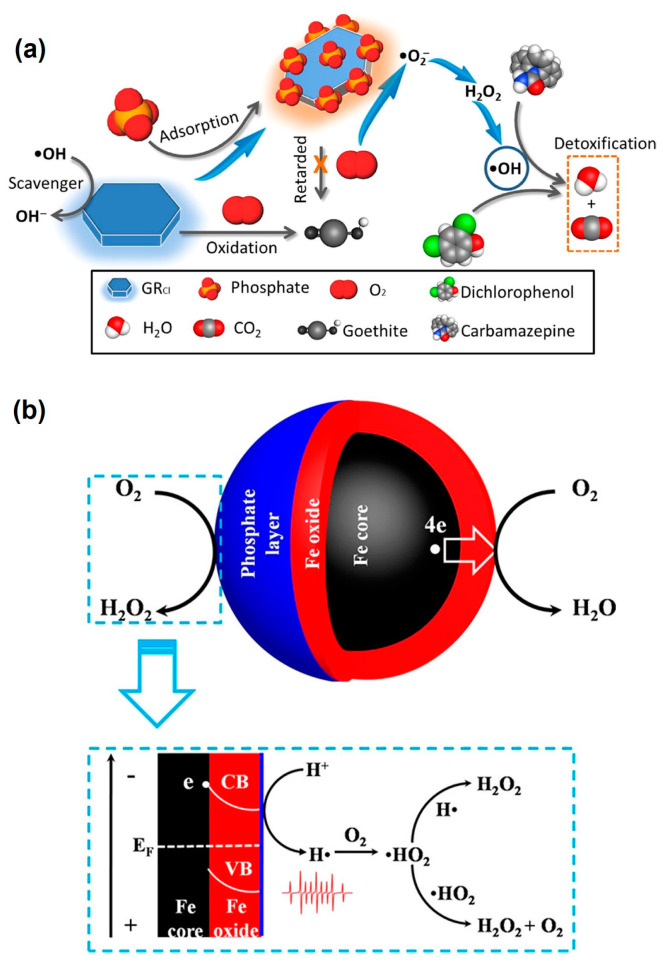
(**a**) Illustration of phosphate-enhanced O_2_ activation by green rust [204]. (**b**) Illustration of phosphate-enhanced O_2_ activation in Fe@Fe_2_O_3_ system [205].

Inorganic anions are prevalent in the subsurface environment and play a significant role in influencing the oxidation behavior of Fe-based materials, mainly by inducing aggregation, increasing the hydrodynamic diameter, and competing with ROS [110]. Studies have shown that the inhibitory effects of common inorganic anions on O_2_ activation by Fe-based materials, such as S–nZVI and nZVI, can be ranked in the order of Cl^−^ < NO_3_^−^ < SO_4_^2−^ < HCO_3_^−^ < HPO_4_^2−^. This discrepancy could be primarily attributed to the varying degrees of competition that these anions exhibit with ROS [203]. Moreover, for Cl^−^, its reaction with •OH to generate secondary chlorine radicals, such as Cl^•^, Cl_2_^•−^, and ClOH^•−^ (Table 8), can partially mitigate the negative effects caused by •OH consumption. The impact of these inorganic anions on the degradation of organic pollutants also depends on the concentrations of the anions and ROS. For a FeS suspension exposed to air, 5 mg/L of Cl^−^ significantly hinders •OH generation. However, with sufficient O_2_ (e.g., through O_2_ purging of the suspension), even 500 mg/L of Cl^−^ has a limited effect on •OH concentration in the suspension due to the abundance of ROS involved in the •OH generation process. Interestingly, the addition of 50,000 mg/L Cl^−^ has been reported to enhance •OH generation, likely due to the production of secondary chlorine radicals [144]. Particularly noteworthy is the effect of the orthophosphate ion (PO_4_^3−^), which could adsorb on the surface of Fe minerals (e.g., green rust) and form the [FeII(OH)_2_-PO_4_]^3−^ complex (Figure 5a), facilitating O_2_ activation and the generation of O_2_^•−^ [204]. When PO_4_^3−^ is introduced to an aerated suspension of surface-oxidized ZVI (Fe@Fe_2_O_3_), the PO_4_^3−^ ions can change the O_2_-reduction pathway from a four-electron to a one-electron process (Figure 5b) [205]. Moreover, the surface phosphate layer induces the in situ generation of atomic hydrogen (•H) on the Fe@Fe_2_O_3_ surface, which can further promote the sequential one-electron O_2_-reduction pathway (Figure 5b) [205].

### 3.4. Microbial Activity

Microbially mediated iron redox reactions are crucial geochemical processes in the environment [208]. Although the presence of O_2_ can lead to a 38–64% decrease in the abundance of iron-reducing bacteria, these microorganisms can recover to 121–793% of their original levels after the restoration of anoxic conditions [209]. Therefore, the role of microorganisms in redox dynamics should garner more attention. Iron-reducing microorganisms could enhance the cycle of Fe(II)/Fe(III) via direct electron transfer [210], serving as extracellular electron shuttles [211] and releasing reduced species (e.g., flavins) [212,213], thus leading to a continuous supply of Fe(II) and a higher ROS yield. Notably, the extent of Fe(III) reduction is related to the mineral composition. For example, goethite with lower crystallinity is preferentially reduced compared to illite by *Shewanella putrefaciens* CN32, a metal-reducing bacterium [213]. Sulfate-reducing microorganisms also have important effects on the iron cycle [214]. Additionally, the presence of certain bacteria, such as neutrophilic iron-oxidizing bacteria [215], can alter the surface properties of Fe minerals and result in the renewal of mineral surfaces through continuous oxidative dissolution [216]. This microbially mediated transformation of iron minerals has significant impacts on O_2_ activation by these minerals.

## 4. Conclusions and Perspectives

Iron-based materials have demonstrated significant potential in activating O_2_ for in situ remediation of sites with organic contaminants. This review systematically examines the current research on O_2_ activation by ZVI, iron sulfide, iron oxide, and Fe-bearing clay minerals. Notably, we have summarized recent findings about the roles of Fe-bearing components in natural soils and sediments for O_2_ activation and ROS generation. The mechanisms for O_2_ activation by these Fe-based materials are thoroughly discussed, including the active sites/species, electron-transfer pathways, and transformation of the materials, and the environmental and operational factors influencing O_2_ activation and ROS generation are analyzed. Despite these significant advances, O_2_ activation by Fe-based materials has not yet been applied for in situ remediation of organic-contaminated sites. Further investigations are needed to address the potential limitations of this promising remediation technology and overcome the barriers to its real-world application:

1. The ROS-generation dynamics under environmental conditions need thorough elucidation and characterization to achieve more accurate prediction and precise control of pollutant removal performance in practical applications. Current studies have demonstrated that the major reactive species generated in O_2_ activation by Fe-based materials are H_2_O_2_, •OH, and O_2_^•−^. However, the potential contribution of other reactive species, especially ^1^O_2_, should be further explored, which has shown tremendous potential in selective oxidation of various contaminants [217,218]. Meanwhile, high-resolution monitoring of ROS-generation dynamics in actual subsurface environments is indispensable, which requires further exploration of novel tools suitable for in situ analysis of trace-level ROS. A notable example of such analytical tools is flow-injection chemiluminescence analysis, which can be performed with a portable device, achieving on-site quantification of •OH in environmental matrices [219].
2. While O_2_ activation by Fe-based materials can degrade a variety of organic pollutants (e.g., TCE, PAHs, phenols, organic dyes, and antibiotics), future efforts are needed to explore its potential for degrading recalcitrant emerging pollutants (e.g., PFASs). The configuration of surface iron sites and interfacial microenvironment significantly affect the efficiency of O_2_ activation. Further research is needed to elucidate the relationship between the functional groups of pollutants and the electron-shuttle mechanism for the tailored development of efficient Fe-based materials for the removal of emerging pollutants. For example, •OH is ineffective in degrading PFASs, whereas O_2_^•−^ has demonstrated the capability to degrade perfluorocarboxylic acids with varying chain lengths [220]. Although O_2_^•−^ is an easily formed intermediate during O_2_ activation by Fe-based materials, it is quickly converted to other ROS. Therefore, nanotechnology-enabled rational material design is needed to manipulate the generation and consumption pathways of O_2_^•−^ during O_2_ activation and improve its selectivity toward reaction with PFASs. This can benefit from theoretical simulations of the interaction between the material surface and O_2_/pollutant molecules under environmentally realistic conditions. Furthermore, the rational design of Fe-based materials for controllable O_2_ activation and ROS generation can be substantially expedited by incorporating machine-learning analysis of large datasets on the structure–reactivity relationships [221,222].
3. Attention should be directed toward conducting pilot-scale applications of this technology to validate its effectiveness in real-world scenarios. In particular, for remediating contaminated sites lacking reactive Fe minerals, it is necessary to introduce Fe-based materials capable of efficient O_2_ activation. Iron-based materials that show excellent performance in laboratory studies may not work when applied in real aquifers, and it is vital to ensure that mass-produced remediation agents exhibit activity comparable to those tested in the initial research and development (R&D) stage. Moreover, the effective delivery of these Fe-based remediation agents can be a bottleneck for ISCO remediation via Fe-mediated O_2_ activation. This calls for a simultaneous evaluation of the transport properties of the Fe-based materials while optimizing their O_2_ activation efficiency.
4. In addition to the above technical challenges, other barriers to the real-world applications of O_2_ activation for site remediation need to be overcome. To be economically viable and competitive, costs associated with the materials, equipment, and power need to be lowered. While Fe is an earth-abundant element, the R&D and scale-up production of sophisticated Fe-based materials still may be costly. Moreover, despite the abundance and availability of O_2_ in the air, the energy required to deliver it into deep aquifers adds to the total cost of this technology. Finally, since Fe-based materials (e.g., nZVI) have shown toxicity to a variety of soil organisms [223,224,225], it is critical to evaluate the potential environmental impact of these materials before they can be safely applied in the subsurface environment. A comprehensive consideration of these factors is warranted to ensure the successful utilization of O_2_, a green and inexhaustible oxidant, for sustainable in situ remediation of organic-contaminated sites.

## Figures and Tables

**Table 1:** Degradation of organic pollutants by Fe-based materials via different mechanisms.

Pollutant	Material	Reaction Mechanism	Reference
Trichloroethylene	nZVI	Reductive dechlorination	[48]
Trichloroethylene	mZVI	Reductive dechlorination	[49]
Trichloroethylene	S-nZVI	Reductive dechlorination	[50]
Trichloroethylene	S-mZVI	Reductive dechlorination	[49]
Trichloroethylene	Fe*_x_*N	Reductive dechlorination	[51]
Chloroform	S–N(C)–ZVI	Reductive dechlorination	[52]
Florfenicol	S-nZVI	Reductive dehalogenation	[53]
Fluorenone	Fe_3_O_4_	H_2_O_2_ activation	[54]
Trichloroethylene	Reduced nontronite	H_2_O_2_ activation	[55]
Diethyl phthalate	Reduced nontronite	H_2_O_2_ activation	[56]
Trichloroethylene	FeS	CaO_2_ activation	[57]
Sulfanilamide	FeS_2_	CaO_2_ activation	[58]
Phenanthrene	FeCo-BDC	Peroxymonosulfate activation	[59]
Perfluorooctanic acid	Fe/AC	Persulfate activation	[60]
Trichloroethylene	nZVI	Persulfate activation	[61]
Ciprofloxacin	FeS_2_	Persulfate activation	[62]
Bisphenol A	Fe_3_S_4_	Peroxymonosulfate activation	[63]
Bisphenol AF	FeS	Periodate activation	[64]
Tetracycline	ZVI	Peracetic acid activation	[65]
Sulfamethoxazole	FeS	Peracetic acid activation	[66]

Note: ZVI, zero-valent iron; nZVI, nanoscale ZVI; mZVI, microscale ZVI; S-nZVI, sulfidated nZVI; S-mZVI, sulfidated mZVI; Fe*_x_*N, iron nitrides; S–N(C)–ZVI, ZVI treated by nitridation and sulfidation; BDC, bimetallic metal-organic frameworks; AC, activated carbon.

**Table 2:** Redox potential of different Fe species.

Species	Redox Potential (V)	Reference
Fe^0^/Fe(II)	−0.44 (vs. SHE)	[80]
Structural Fe(II)/Fe(III) of pyrite	0.66 (vs. SHE)	[81]
Structural Fe(II)/Fe(III) of clay mineral	−0.6 to +0.6 (vs. SHE)	[82]
Fe(III)/Fe(II)-CA	0.37 (vs. NHE)	[83]
Fe(III)/Fe(II)-OA	0.002 (vs. NHE)	[83]
Fe(III)/Fe(II)-EDTA	0.12, 0.11, and 0.096 (vs. NHE)	[83]
Fe(III)/Fe(II)-EDDS	0.19 (vs. NHE)	[83]
Fe(III)/Fe(II)-NTA	0.10 and 0.39 (vs. NHE)	[83]
Fe(H_2_O)_6_^2+^/Fe(H_2_O)_6_^3+^	0.77 (vs. NHE)	[83]

Note: SHE, standard hydrogen electrode; NHE, normal hydrogen electrode; CA, citrate; OA, oxalate; EDTA, ethylenediaminetetraacetic acid; EDDS, N,N′-1,2-ethanediylbis-1-aspartic acid; NTA, nitilotriacetic acid.

**Table 3:** Degradation of organic pollutants via O_2_ activation by ZVI-based materials.

Material	Pollutant	Removal Ratio (%)	Reaction Time (h)	pH	Rate Constant	Reference
ZVI	EDTA	100	2.5	6.0 ± 0.2	1.02 h^−1^	[68]
nZVI	2-Chlorobiphenyl	59.4	4	5.0	0.0035 min^−1^	[84]
S-nZVI	Bisphenol A	100	6	5.0	59.2 ± 2.29 h^−1^	[85]
Fe@Fe_2_O_3_	4-Chlorophenol	77.8	7	6.0	0.22 h^−1^	[89]
Al–Fe	4-Chlorophenol	43.7	5	2.5	N/A	[91]
Fe/Cu	4-Chlorophenol	100	2	3.0	N/A	[92]
Fe/Cu	Diclofenac	96	2	6.0	N/A	[93]
Mg/Fe	4-Chlorophenol	100	0.75	3.0	N/A	[94]
Fe/Mn	Enrofloxacin	100	1	3.0	N/A	[95]
ZVI	Enrofloxacin	58.6	1	3.0	N/A	[95]
S-nZVI	p-Nitrophenol	99.3 *	2	7.6	0.769 min^−1^	[96]
mZVI/NGB	Tetracycline	100	0.83	5.8	N/A	[97]
3D-GN@nZVI	Sulfadiazine	81.0	2	3.0	N/A	[98]
Cu/Fe-BC	Ciprofloxacin	93.2 *	1.5	5.0	0.052 min^−1^	[99]
Cu/Fe-BC	Enrofloxacin	88.9 *	1.5	5.0	0.036 min^−1^	[99]
Cu/Fe-BC	Norfloxacin	95.4 *	1.5	5.0	0.096 min^−1^	[99]
Cu/Fe-BC	Tetracycline	82.3 *	1.5	5.0	0.037 min^−1^	[99]
Cu/Fe-BC	Methylene blue	95.6 *	1.5	5.0	0.145 min^−1^	[99]
ZVI-BC	Tetracycline	93.1	6	Unadjusted	N/A	[100]
Zn-Fe-CNTs	Sulfamethoxazole	95.3	0.33	1.5	N/A	[101]
Zn-Fe-CNTs	4-Chlorophenol	90.8	0.33	2.0	N/A	[102]
nZVI@MSN	Nitrobenzene	96.5	0.33	3.0	0.201 min^−1^	[103]

Note: N/A, data not available. *, data obtained from the literature using the Getdata 2.26 software.

**Table 5:** Degradation of organic pollutants via O_2_ activation by iron (oxyhydr)oxides.

Material	Pollutant	Removal Ratio (%)	Reaction Time (h)	pH	Rate Constant	Reference
Magnetite	2-Chlorobiphenyl	80	4	3.0	N/A	[149]
Cu^0^/Fe_3_O_4_	4-Chlorophenol	99.5	1	7.0	0.073 min^−1^	[151]
Zn^0^-CNTs-Fe_3_O_4_	4-Chlorophenol	99	0.33	1.5	N/A	[152]
CNTs-Fe_3_O_4_	4-Chlorophenol	25	0.33	1.5	N/A	[152]
b-CoS_2_/Fe_3_O_4_	4-Nitrophenol	62.3 *	0.25	5.0	N/A	[153]
b-CoS_2_/Fe_3_O_4_	Methyl orange	85.7	0.25	5.0	N/A	[153]
b-CoS_2_/Fe_3_O_4_	Sulfadiazine	67.1	0.25	5.0	N/A	[153]
b-CoS_2_/Fe_3_O_4_	Tetracycline	96.0	0.25	5.0	N/A	[153]
b-CoS_2_/Fe_3_O_4_	Rhodamine b	98.6 *	0.25	5.0	N/A	[153]
b-CoS_2_/Fe_3_O_4_	Malachite green	91.5 *	0.25	5.0	N/A	[153]
Ferrihydrite	Phenol	29.8 *	10	7.0	N/A	[154]

Note: N/A, data not available. *, data obtained from the literature using the Getdata 2.26 software.

**Table 7:** Stability constants of Fe(II)/Fe(III)-complexes with common organic ligands and rate constants for reaction of these ligands with •OH.

Species	Log β of Fe(II)-Complex	Log β of Fe(III)-Complex	Reaction Rate Constants with •OH (M^−1^ s^−1^)	Reference
EDTA	14.3	25.1	2.0 × 10^9^	[83]
EDDS	N/A	20.6	2.5 × 10^9^	[83,187]
NTA	8.05	15.90	5.5 ×10^8^	[83,186]
CA	3.2	8.36–12.38 and 11.5	3.2 × 10^8^	[83]
OA	> 4.70	9.4	1.0 × 10^7^	[83]
HA	N/A	6.65–7.59	N/A	[83]

Note: N/A, data not available.

**Table 8:** Reaction rate constants between •OH and inorganic anions.

Reaction	Rate Constant (M^−1^ s^−1^)	Reference
Cl^−^ + •OH → ClOH^•−^	4.3 × 10^9^	[206]
ClOH^•−^ → Cl^−^ + •OH	6.1 × 10^9^	[206]
ClOH^•−^ + H^+^ → Cl^•^ + H_2_O	4.3 × 10^10^	[206]
Cl^•^ + Cl^−^ → Cl_2_^•−^	1.0 × 10^5^	[206]
•OH + HCO_3_^−^ → CO_3_^•−^ + H_2_O	8.5 × 10^6^	[207]
•OH + CO_3_^2−^ → CO_3_^•−^ + OH^−^	3.9 × 10^8^	[206]
HPO_4_^2−^ + •OH → HPO_4_^•−^ + OH^−^	8.0 × 10^5^	[206]

## Data Availability

No new data were created or analyzed in this study. Data sharing is not applicable to this article.
